# Systematic literature review on surgical site preparation in paediatric surgery

**DOI:** 10.1186/s12887-022-03502-z

**Published:** 2022-07-28

**Authors:** Isabella Bielicki, Ulrike Subotic, Julia Anna Bielicki

**Affiliations:** 1 Department of Paediatric Surgery, University Children’s Hospital Basel, Spitalstr. 33, 4056 Basel, Switzerland; 2 Department of Paediatric Surgery, University Children’s Hospital Basel, Basel, Switzerland; 3 Division of Infectious Diseases and Hospital Epidemiology, Paediatric Pharmacology Group, University Hospital Basel, Basel, Switzerland University of Basel Children’s Hospital, Basel, Switzerland National Centre for Infection Prevention, SwissNoso Bern, Switzerland

**Keywords:** Review, Systematic review, Anti-infective agents, local/therapeutic use, Child, Surgical wound infection/prevention & control

## Abstract

**Background:**

Surgical site infections (SSIs) in children represent a common and serious postoperative complication. Surgical skin preparation is an essential preventive measure in every surgical procedure. The most commonly used antiseptic agents for surgical skin preparation are chlorhexidine gluconate and iodophors in alcohol-based solutions. In adult patients the use of chlorhexidine-containing antiseptic solutions for preoperative skin preparation has been advocated to reduce SSI rates. Our objective was to conduct a systematic literature review on use of antiseptic agents for surgical skin preparation in children less than 16 years of age.

**Methods:**

A systematic review of MEDLINE, EMBASE, CINAHL and CENTRAL was performed using both MeSH and free text terms and using the relevant Cochrane filter to identify full text randomized trials (RCTs) and comparative observational studies. Interventions of interest were the choice of main agent in antiseptic solutions (chlorhexidine/povidone-iodine/alcohol) compared with each other or with other antiseptic agents. Primary outcome was the reported rate of surgical site infections.

**Results:**

In total 8 studies were included in the review; 2 RCTs and 6 observational studies. Observational studies generally did not primarily investigate the association of different antiseptics with subsequent SSI. The identified randomised controlled trials included only 61 children in total, and were of low quality. Consequently, we did not conduct a formal meta-analysis. Since the publication of a comprehensive systematic review of perioperative measures for the prevention of SSI in 2016, no randomized controlled trials comparing antiseptic agents for surgical skin preparation in paediatric surgery have been conducted.

**Conclusion:**

Robust evidence on the optimal skin antisepsis to reduce SSIs in children is lacking. Direct extrapolation of effects from trials involving adults is not appropriate as physiologic characteristics and risk factors for SSIs differ between adults and children. It is therefore essential to conduct high quality RCT investigating interventions to identify optimal measures to reduce SSI rates in children.

**Trial registration:**

Prospero registration (CRD42020166193).

**Supplementary Information:**

The online version contains supplementary material available at 10.1186/s12887-022-03502-z.

## Background

Postoperative surgical site infection (SSI) may be associated with any type of surgical procedure and is a common and serious postoperative complication. SSIs are among the most common hospital acquired infections in children and represent a relevant burden to patients and their families as well as health care systems [1]. With about one quarter of cases SSI is the leading cause for readmissions among children undergoing surgery [2]. According to the World Health Organization (WHO) up to 50% of SSIs could be prevented [3]. Prevention intervention bundles including skin antisepsis have been successfully implemented to reduce SSI rates in children [4]. Although SSI rates in children are lower compared to adult population (2% in children versus 45% in adults after colorectal surgery) [5], the cost of each infection is high at 2000 € per infection [6]. In high-income countries such as the U.S. on average 3.9 million surgical procedures in children and adolescents are performed each year [7]. Assuming a rate of SSI of 2% this results in at least 78,000 cases of SSI each year, not accounting for patient groups and surgical procedures with a significantly higher incidence. Neonates and children with congenital anomalies are known to be especially vulnerable [8–10]. Incidence of SSI in this patient group has been reported as high as 17% [8]. The burden of SSIs is even higher in low- and middle-income countries (LMICs) at 24.7% compared with 6.3% in high-income countries [11]. Against this background and the estimated number of 1.7 billion children per year in LMIC in need of surgery, prevention of SSIs is an essential part of improving health care for children globally [12].

Based on multiple systemic literature reviews, the World Health Organization (WHO) [13, 14] as well as the National Institute for Health and Care Excellence (NICE) [15] and the Asia Pacific Society of Infection Control (APSIC) [16] have recently published guidelines on the prevention of SSIs. The majority of literature on SSI prevention reports on data from adult patients. For specific procedures with a high risk of SSI in children, such as in cardiovascular and neurosurgery, it has been shown that intervention bundles can reduce the rate of SSI [17–23]. Nonetheless, there is little evidence on the influence of optimal individual preventive measures, for example surgical skin preparation agents, in children.

Surgical skin preparation is performed in order to reduce bacterial load at the surgical site. The most widely used agents are chlorhexidine gluconate (CHG) and iodophors (i.e. povidone-iodine (PVP)) in alcohol based solutions [24]. While other interventions, such as the use of antiseptic-coated sutures or whole-body washing, are also of interest, surgical skin preparation is particularly relevant as being part of every surgical procedure independent of circumstances (elective vs. emergency procedure), comorbidities and age. Worldwide, antiseptic solutions are expected to be readily available and application is simple and can be carried out without specific training. The literature review conducted by the WHO in November 2016 recommends the use of CHG solutions for adult surgery [14]. Nonetheless, as has been shown in surveys conducted among paediatric surgery units in Great Britain [25], Germany and Switzerland there is no standardization in choice of antiseptic agent for surgical skin preparation in children. Less than 50% of questioned surgeons use CHG in neonates and preterm babies. Although newer evidence suggests both CHG and PVP to be safe alternatives for surgical skin preparation in infants [26], concerns for side effects such as local or systemic toxicity persist [24, 27]. Especially in preterm neonates < 34 weeks and in very low birth weight infants, a patient group particularly at risk of SSI, evidence on safety profiles on antiseptic solutions is equivocal [24].

In response to the lack of evidence in the choice of antiseptic solution for surgical skin preparation in paediatric surgery pointed out by the WHO, we aimed to systematically review the current body of evidence.

## Methods

A systematic review of the published literature was conducted and is reported in accordance with Preferred Reporting Items for Systematic Reviews and Meta-Analyses (PRISMA). A protocol for this review was prospectively registered with PROSPERO (CRD42020166193).

### PICOS (Participants, intervention, comparator, outcomes and study design)

Studies evaluating the interventions of interest in children under 16 years of age undergoing any surgery, including minimally invasive surgery, elective and emergency surgery, were included. Studies of surgical procedures that did not include a visible incision and therefore did not result in the presence of a conventional surgical wound or did not require suturing or closure of the wound were excluded. Studies which included both adults and children were excluded, if the age of included children in trial was ≥ 16 years of age. Interventions and comparators studied were: choice of main agent in antiseptic solutions (chlorhexidine/povidone-iodine/alcohol) compared with each other or with other antiseptic agents. The primary outcome of interest assessed is the SSI rate. All types of SSIs were considered (superficial, deep wound, organ space). Randomized controlled trials (RCTs) and comparative observational studies were considered eligible for inclusion.

### Search strategy

Electronic searches were conducted on Feb 29, 2020 and on Dec 8, 2020 using OVID SP on the following databases: MEDLINE (1946-Dec week 4 2020), Excerpta Medica Database (EMBASE) (1974–2020 Dec 8th); Cumulative Index to Nursing and Allied Health Literature (CINHAL) (1988–2019) and Cochrane Central Register of Controlled Trials (CENTRAL). No restrictions on publication language were applied. A comprehensive list of search terms was used, including Medical subject Headings (MesH) (see Additional File 1). References from relevant articles were identified by using the search terms “surgical wound infection”, “surgical site infection”, “SSI”, “child*”, “peadiat*”, “pediat*”, “chlorhexidine*”, “alcohol*”, “ethanol”, and “iodin*”. References were then screened by titles and abstract in order to find relevant studies. The full text of all potentially eligible articles was obtained. Duplicate studies were excluded. Full text articles were screened for eligibility based on the prespecified eligibility criteria. Data was extracted from the selected articles and converted into a tabulated form, including study year, study design, study time, surgical procedure or degree of contamination, mean age, number of patients, type of antiseptic agent, criteria for diagnosis of SSI and SSI rate, duration of follow-up and rate of preoperative prophylactic antibiotic therapy. Quality of RCTs was graded using the van Tulder scale [28]. Quality of the observational studies was graded using the Newcastle–Ottawa Scale [29]. As preliminary literature searches already showed little evidence in paediatric patients no plans for meta-analysis were made before conducting the search. Overall level of evidence was assessed according to Oxford Centre for Evidence-Based Medicine [30].

## Results

**Fig. 1:**
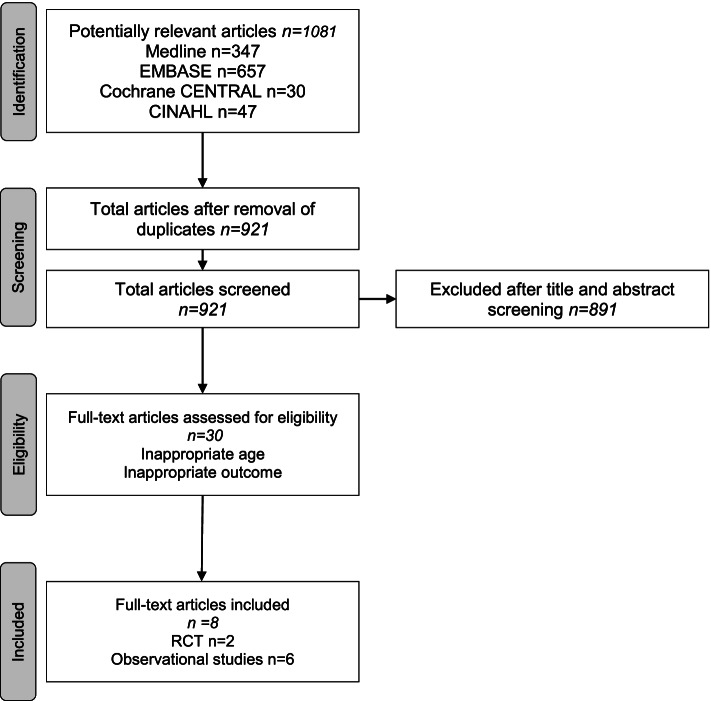
PRISMA flow diagram of search results

The search identified 991 records (Fig. 1). After removal of duplicates, 885 articles were screened. 855 articles were excluded after screening of titles and abstracts. Thirty full texts were assessed for eligibility. Of those twenty-two were excluded, mostly because of inappropriate age or inappropriate outcome. A total of eight articles remained, of which 2 were RCTs [31, 32] and 6 were observational studies [9, 33–37].

### Study characteristics

Study characteristics are summarized in Tables 1 and 2. Two single centre RCTs comparing povidone-iodine with methyl-alcohol and chlorhexidine respectively were identified in the search. Both trials included children as well as adults. Of the six observational studies identified in the search process, two included adults as well as children, one included only neonates and three included children of different age groups. Surgical wounds and procedures were mostly classified as clean or clean contaminated. Only Lubega et al.also incorporated surgical procedures involving dirty or infected wounds [37].

**Table 1:** Study characteristics of included RCTs

Study	Meier et al. (2001) [38]	Berry et al. (1982) [31]
Number of patients	53 (≤ 13 years)	8 (< 15 years)
Number of centres	1	1
Dates	Jun 1994- Dec 1995	May 1978- Feb 1980
Surgical classification	Clean	Clean and clean-contaminated
Mean age	Not stated	Not stated
Intervention	Povidone-iodine vs soap and methyl alcohol	Povidone-iodine vs. chlorhexidine
SSI criteria	Redness of wound or purulent discharge	Redness of wound, oedema or purulent discharge
Duration of follow-up	30 days	Min 3–4 days, until discharge
Routine prophylactic antibiotics	no	Colonic and rectal surgery

**Table 2:** Study characteristics of included observational studies

Study	Lubega et al. (2017) [37]	Chang et al. (2011)	Rojo et al. (2012) [35]	Bashyal et al. (2009) [34]	Mc Cray et al. (1986) [33]	Bucher et al. (2011) [42]
Study design	Prospective	Prospective	Retrospective case–control	Retrospective	Retrospective case–control	Retrospective case–control
Dates	Sep 2014- Jan 2015	Jan-Oct 2007	Oct 2010-Jan 2012	Jan 1994- Dec 2005	Jan-Aug1983	Jan 1996- Dec 2007
Type of surgery	Emergency surgery	Reimplantation of bone flaps after craniotomy/craniectomy	Neonatal surgery	Pinning of supracondylar fracture	Genitourinary reconstructive surgery	Clean and clean-contaminated procedures
Number of patients	Adults and children *n* = 114	Adults and children *n* = 373	Neonates *n* = 90	Children *n* = 622	Boys *n* = 70	Children *n* = 477
Age	IQR 29 years	Mean 48 years	Mean 32.5 gw	Not stated	Mean group SSI 9.5 years, Mean non-SSI 5.2 years	11% neonates, 27% infants, 43% children, 18% adolescents
Antiseptic agents used	Iodine, Chlorhexidine (number of patients per group not stated)	10% PVP gel & solution (*n* = 230), 10% PVP gel (*n* = 116), CHG (*n* = 27)	Betadine (*n* = 14), CHG (*n* = 70), other (*n* = 6)	PVP wash before reduction (*n* = 6), Alcohol wash & PVP spray after reduction (*n* = 110), PVP paint after reduction (*n* = 65), PVP spray after reduction (*n* = 278)	1% Hexachlorophene & 70% Alcohol/aceton & Benzalkonium chloride (*n* = 21), PVP or Hexachlorophene (*n* = 49)	Iodine (*n* = 431), Chlorxylenol (n = 38), CHG (*n* = 1), Alcohol (*n* = 3)
SSI criteria	Not stated	CDC criteria	CDC criteria	Not stated	Presence of fever and purulent drainage	CDC criteria
SSI rate	16.4% (18/110)	5.8% (22/377)	44.4% (40/90)	1% (6/622)	11.4% (8/70)	0.99% (159/16031)
Difference between antiseptic agents	n.s	PVP gel & solution superior (OR 0.21, *p* = 0.04)	n.s	n.s	n.s	n.s
Duration of follow-up	Max. 30 days postoperatively	30 days	30 days	Min. 2 weeks after pin removal	n.s	30 days
Routine prophylactic antibiotics	n.s	n.s	84% (76/90)	26% (163/622)	All patients	32% (155/477)

### Randomized controlled trials

Meier et al. [38] conducted a single-centre randomized, controlled, open label trial comparing market soap and methyl alcohol with povidone-iodine for preoperative skin preparation with 200 participants of all ages undergoing elective inguinal hernia repair in a developing world country hospital (53 children less than 13 years). It is not reported which type of solution was used (aqueous vs. alcoholic, concentration of povidone iodine). Demographic characteristics in the paediatric group were not reported. The infection rate in children was lower compared to adults, although not significantly so (1.9% vs. 6.5%; *p* = 0.294). The infection rate between trial arms did not differ significantly (methyl alcohol group 5.1% vs. povidone iodine group 5.9% SSI; *p* = 1.000). Berry et al. [31] also conducted a single-centre randomized, controlled, open label trial comparing povidone-iodine and chlorhexidine both for surgical scrub (povidone –iodine 10% in alcohol or chlorhexidine 0.5% in alcohol) and skin preparation (7.5% povidone-iodine or 0.5% chlorhexidine in alcohol) in 866 patients. The trial was conducted at Western, General Hospital in Edinburgh between May 1978 and February 1980 and all patients undergoing elective surgery were included in the trial. Surgical procedures were either clean non-abdominal operations or clean/clean-contaminated abdominal or genitourinary interventions. Only eight children less than 15 years of age were recruited. Most likely, because of the small number of children included in the trial, the authors did not further differentiate between adult and paediatric patients. Between trial arms (scrub and skin preparation with povidone-iodine vs. chlorhexidine) there was a significant difference in SSI rate (14.8% PVP vs. 9.8% CHG; *p* = 0.03) at the time of the patients ‘ discharge after the index surgery. Nevertheless, as there were more infections with CHG compared to PVP in some subgroups, i.e. larger bowel surgery, hernia repair and genitourinary surgery, the authors conclude that there is no clear benefit of CHG over PVP for surgical scrub and skin preparation.

### Observational studies

Two prospective and four retrospective observational studies [9, 33–37] were identified in the literature search (Table 2). Of the included studies only one was conducted in a LMIC. Lubega et al. [37] prospectively investigated the rate and risk factors of SSI after emergency surgery in a regional referral hospital in Uganda. All degrees of wound contamination were included and skin antiseptic agents used were PVP and CHG. Only eight children were included in the trial. Further analysis of the paediatric subgroup was not conducted. Overall, multivariate analysis did not show a significant difference between antiseptic agents used. There was only one further observational study recording the incidence of SSIs prospectively. Chiang et al. [36] investigated the clinical significance of positive cranial bone flap cultures and the associated risk of SSI after craniotomies or craniectomies. Both adults and children were included in the study. Unfortunately, the authors do not state how many paediatric patients were included. PVP solution and gel as well as CHG were used for preoperative skin preparation. Although application of PVP gel and solution resulted in significantly less SSI, the authors emphasize that this result may be biased due to the lack of data for some procedures and change of skin preparation policy during the study period. Four retrospective observational studies [9, 33–35] investigating risk factors for SSIs in children were identified in the literature search (Table 2). One of these exclusively investigated risk factors in neonates. Rojo et al. conducted a case–control study of 90 surgical in neonates with a mean age of 32.5 gw. CHG was the antiseptic agent used in most cases (PVP (*n* = 14), CHG (*n* = 70), other (*n* = 6)). Neither in this study nor in the other observational studies any significant differences between antiseptic agents used were identified.

### Quality of evidence

The quality of the included RCTs was graded using the van Tulder scale [28] (Table 3) and Newcastle–Ottawa Scale [29] for observational studies (Table 4). It has to be noted, that some of the studies [31, 33]included in this review have been conducted in the 80ies. At this time infection control strategies were not yet implemented widely. There have been significant changes in outcome definitions as well as changes in products and surgical methodology since. Therefore, transferability of results is limited. The trial on preoperative skin preparation conducted by Meier et al. [38] only included 53 paediatric patients with an unusual cut-off age for paediatric patients (less than 13 years). It is not clear what the mean age in this group of patients was. Moreover, subgroup analysis was not carried out, due to low rate of wound infections in this group. As the trial was conducted in a LMIC with the goal to identify cost-effective measures to reduce SSI rates the selection of antiseptic agents is not representative for general paediatric surgery. Berry et al. [31] compared the use of PVP and CHG for surgical scrub and skin preparation. As the authors point out it is not clear whether differences in outcome are due to one or both interventions. Furthermore, only 1% of patients recruited can be considered paediatric. Thus, the significance of the results for this patient group is highly questionable. The observational studies identified in the search included a multitude of different antiseptic agents for surgical skin preparation. All studies included iodine-based solutions for surgical skin preparation. Four of six studies included CHG as alternative antiseptic agent. Overall patients were not allocated evenly to either surgical skin preparation with iodine or CHG (see Table 2). Furthermore, degree of contamination, type of surgical procedures and rate of prophylactic antibiotic therapy were not comparable among studies. All in all, the quality of evidence of included RCTs and observational studies was low to moderate and was not sufficient in order to conduct a meta-analysis. Level of evidence of the studies according to Oxford Centre for Evidence-Based Medicine was low (see Table 5) and does not allow for a recommendation of choice of antiseptic for preoperative skin preparation above level C [30].

**Table 3:** Quality assessment of observational studies by modified Newcastle–Ottawa quality assessment scale

	Item									
Study	Selection				Comparability ^**a**^	Outcome/Exposure				Power
	Cohort studies									
	Representativeness of the exposed cohort (Maximum★)	Selection of non-exposed cohort (Maximum★)	Ascertainment of exposure (Maximum★)	Demonstration that the current outcome of interest was not present at the start of the study (Maximum★)	Comparability of cohorts on basis of the design analysis (Maximum★★)	Assessment of outcome (Maximum★)	Length of follow-up (Maximum★)	Adequacy of follow-up (Maximum★)	Total score (out of nine)	
Lubega et al. (2017) [37]	★	★	★	-	-	-	★	-	4	Underpowered
Chang et al. (2011)	★	★	★	-	-	★	★	-	5	Underpowered
Bashyal et al. (2009) [34]	★	★	★	-	-	-	-	-	3	Underpowered
	Case control studies									
	Adequate case definition (Maximum★)	Representativeness of the cases (Maximum★)	Selection of controls (Maximum★)	Definition of controls (Maximum★)	Comparability of cohorts on basis of the design analysis (Maximum★★)	Ascertainment of exposure	Same method of ascertainment for cases and controls	Non-Response rate	Total score (out of seven)	
Rojo et al. (2012) [35]	★	★	★	★	★★	★	★	-	8	Underpowered
Mc Cray et al. (1986) [33]	-	★	★	-	-			-	4	Underpowered
Bucher et al. (2011) [42]	★	★	★	★	★★	★	★	-	8	Underpowered

^a^ A maximum of 2 stars can be allotted in this category. Scores were allocated as follows:

One point was allocated if SSI rate was adjusted for by age. Another point was given for adjusting by degree of contamination or administration of perioperative antibiotic prophylaxis

**Table 4:**
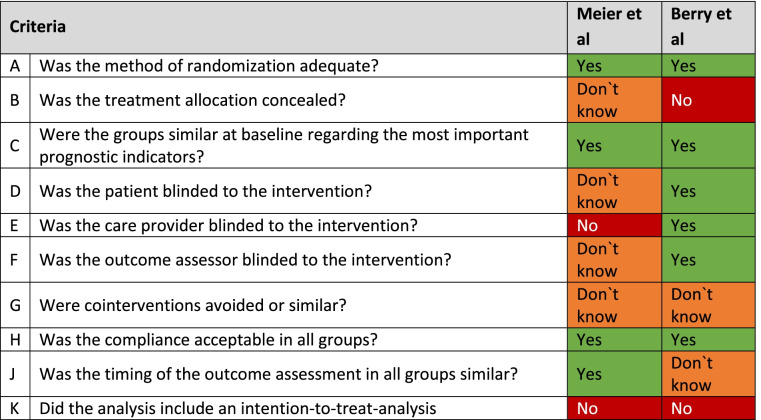
Quality assessment of included studies using van Tulder scale

**Table 5:** Level of evidence according to Oxford Centre for Evidence-Based Medicine

Study	Design	Level of evidence
Lubega et al. (2017) [37]	Prospective cohort study	4
Chang et al. (2011)	Prospective cohort study	4
Rojo et al. (2012) [35]	Retrospective case–control study	3b
Bashyal et al. (2009) [34]	Retrospective cohort study	4
Mc Cray et al. (1986) [33]	Retrospective case–control study	4
Bucher et al. (2011) [42]	Retrospective case–control study	3b
Meier et al. (2001) [38]	Randomized controled trial	2b
Berry et al. (1982) [31]	Randomized controled trial	2b

## Discussion

This systematic literature review confirms the lack of evidence on strategies for surgical skin preparation in children. In particular, there are no data to support the use of specific agents for high-risk groups, such as neonates and especially preterm babies.

Establishing safe and effective surgical care for children is a critical but neglected area within global surgery [7]. Simple preventive measures, such as optimal surgical hand and skin preparation and appropriate antibiotic prophylaxis have shown to significantly lower the rate of SSI independent of available resources and surgical procedure [39, 40]. Implementing and investigating standardized perioperative management programs in children does not only reduce postoperative morbidity and associated health-care costs. Standardized surgical skin preparation is an important part in the framework of antibiotic stewardship programs and as such can support adherence to other integral parts of perioperative management, such as antibiotic prophylaxis. Moreover, as patients experiencing SSI generally require antibiotic treatment, reducing the rate of SSI in children decreases the need for antibiotic treatment postoperatively.

Importantly, evidence from the adult population to paediatric perioperative management is not readily transferable. Although some may argue that school aged children and adolescents may be comparable to adult patients, this is definitely not the case in neonates, infants and toddlers. Skin absorption and fragility, wound healing and skin microbiome change depending on age and environment [41]. Children have a different spectrum of comorbidities, physiological characteristics and therefore also have different risk factors for SSIs [42]. Interventions proven to be effective in adults do not necessarily have the same impact in children and could possibly have unexplored adverse effects [43]. Skin toxicity and systemic absorption have been of special concern for many agents in use in neonatal surgery, such as chlorhexidine, povidone-iodine and alcoholic preparations [24]. Our findings underline the ongoing dilemma on choice of antiseptic agents for skin preparation in children. Not only in paediatric surgery [25], but also in neonatal skin care and before catheter insertion in children a variety of agents and concentrations are in use [44–46]. Ideally antiseptic agents used for skin preparation in children should have an antimicrobial effect, should have a quick onset of action and long residual effect without any or minimal toxic effects on the skin and the organ systems [24]. In adult patients, the latest literature review on the use of antiseptic solutions conducted by NICE in 2019 included 28 RCTs [15]. Like the WHO in 2016 [14], the authors conclude that alcoholic solutions are more effective in reducing the risk of SSIs and that specifically alcohol-based chlorhexidine gluconate has the lowest risk of SSIs occurring postoperatively. Both reviews state that evidence is of moderate to low quality. The RCTs including children in this field are of low quality [31, 38]. The observational studies available did not investigate differences in SSI rate between antiseptic agents as their primary objective [9, 33–37].

Antiseptic solutions have been investigated in other fields of skin antisepsis in children [24]. CHG has been recommended by the WHO for umbilical cord care in community and primary care settings in developing countries [47] and has shown to be superior to other agents in this setting [48]. Daily washings with CHG for critically ill children and application of CHG-containing solutions before central venous catheter insertion are other examples in which CHG has shown to be effective in the paediatric population [49, 50]. All in all, there is substantial evidence suggesting that CHG containing antiseptic skin preparations are effective in paediatric infection prevention and control [24]. However, comparative research to evaluate different agents is extremely rare.

As shown in this review evidence on the optimal choice of antiseptic solutions for preoperative skin preparation in children is scarce and of low quality. Though some may argue that it is lengthy and expensive to conduct randomized trials on perioperative measures in the paediatric population, it is necessary and as demonstrated by Renko et al. [51] possible. This RCT investigating the efficacy of different sutures supports the use of more costly triclosan-coated sutures because of their efficacy in reducing SSIs in this vulnerable population. While the efficacy of skin antiseptics may be comparable and their costs are generally low, their safety profiles may differ, influencing selection. Furthermore, even small efficacy advantages may translate into a large number of SSIs prevented. New innovative designs, such as point-of-care cluster randomization may allow for the efficient yet robust evaluation of perioperative care, including skin antiseptics, aiming to prevent common post-operative complications for paediatric surgical interventions.

## Conclusion

The findings of this study confirm the lack of evidence on choice of antiseptic agent for surgical site preparation in children. Direct transfer of evidence from the adult population to paediatric surgery is not appropriate due to important differences in possible toxic local and systemic side effects, spectrum of comorbidities and type of surgery in children. Therefore, it is essential to conduct high quality RCT investigating interventions to identify optimal measures to reduce SSI rates in children.

## Supplementary Information

**Additional file 1.** 

## Data Availability

The datasets used and/or analysed during the current study are available from the corresponding author on reasonable request.

## Abbreviations

APSIC: Asia Pacific Society of Infection Control
CDC: Centers for Disease Control and Prevention
CENTRAL: Cochrane Central Register of Controlled Trials
CHG: Chlorhexidine gluconate
CINHAL: Cumulative Index to Nursing and Allied Health Literature
EMBASE: Excerpta Medica Database
Gw: Gestational week
IQR: Interquartile range
LMICs: Low- and Middle-income Countries
Max.: Maximal
Min.: Minimal
NICE: National Institute for Health and Care Excellence
n.s.: Not stated
PRISMA: Preferred Reporting Items for Systematic Reviews and Meta-Analyses
PVP: Povidone-iodine
RCT: Randomized controlled trial
SSI: Surgical site infection
U.S.: United States
WHO: World Health Organization
