# Supplementary material for: Systematic literature review on surgical site preparation in paediatric surgery
Source: BMC Pediatr. 2022 Jul 28;22:455. doi: 10.1186/s12887-022-03502-z (PMC9336073; doi:10.1186/s12887-022-03502-z)
Supplement: Supplementary file 1 — Additional file 1. [file 12887_2022_3502_MOESM1_ESM.docx]

Appendix1: Eligibility criteria for study search

| **Inclusion criteria** | - Children < 16 years of age - Undergoing any type of surgery (including minimally invasive surgery, elective and emergency surgery) |
| --- | --- |
| **Exclusion criteria** | - Age ≥ 16 years - Procedures   - not including a visible incision   - not resulting in the presence of a conventional surgical wound   - not requiring suturing or closure of the wound |
| **Interventions/comparators** | - Choice of antiseptic agent for surgical skin preparation (CHG/PVP/alcohol) - Compared with each other or with other antiseptic agents |
| **Primary outcome** | - Rate of surgical site infection (all types) |
| **Types of studies included** | - Randomized controlled trials - Comparative observational studies |

Appendix2: Search strategy

| Database | Search strategy |
| --- | --- |
| Medline (via OVID) | exp Chlorhexidine/ or Chlorhexidin*.ti,ab. or Chlora?rep.ti,ab. or Chlorostat.ti,ab. or Hibi*.ti,ab. or exp Ethanol/ or Ethanol*.ti,ab. or Alcohol*.ti,ab. or IPA.ti,ab. or Soluprep.ti,ab. or Cutasept.ti,ab. or softasept.ti,ab. or exp Povidone-Iodine/ or exp Povidone/ or exp Iodine Compounds/ or iodin*.ti,ab. or iodophor*.ti,ab. or Povidon-Iod*.ti,ab. or Betadona.ti,ab. or Brauno*.ti,ab. or Destrobac.ti,ab. or Jodoplex.ti,ab. or Pol?sept.ti,ab. or Sepso J.ti,ab. or Topionic.ti,ab. or Wundesin.ti,ab. or Betasept*.ti,ab. or exp Antiinfective Agents, Local/ or ((solution or agent) adj2 (antiseptic* or antimicrobial*)).ti,ab. or (skin adj2 (d?sinfectant or srub or preparation* or antisep* or detergent* or cleaning or cleansing)).ti,ab.  AND  exp surgical wound infection/ or (postop* adj2 infection).ti,ab. or (incision* adj2 (infection* or abces*)).ti,ab. or (postop* adj2 absces*).ti,ab. or (surgical adj2 infection*).ti,ab. or SSI*.ti,ab. or wound infection.ti,ab.  AND  exp child/ or exp Infant/ or exp Infant, Newborn/ or exp Adolescent/ or exp Child, Hospitalized/ or exp Intensive Care Units, Pediatric/ or exp Intensive Care Units, Neonatal/ or exp Hospitals, Pediatric/ or exp Intensive Care Units, Pediatric/ or p?ediatric surgery.ti,ab. or (adolescen*or infan* or newborn* or (new adj born*) or neonat* or child* or pediatric* or paediatric* or PICU or NICU or PICUs or NICUs).ti,ab.  AND  exp cohort studies/ or cohort$.tw. or controlled clinical trial.pt. or epidemiologic methods/ limit to yr=1966-1989 or exp case-control studies/ or (case$ and control$).tw. or (case$ and series).tw.  OR  randomized controlled trial.pt. or controlled clinical trial.pt. or randomized.ab. or placebo.ab. or drug therapy.fs. or randomly.ab. or trial.ab. or groups.ab.  not exp animals/ not humans.sh. |
| Embase (via OVID) | exp surgical infection/ or exp wound infection/ or exp postoperative complication/ or ssi.ti,ab. or (surgical adj2 infection*).ti,ab. or wound infection*.ti,ab. or (postop* adj2 infection).ti,ab. or (incision* adj2 (infection* or abces*)).ti,ab. or (postop* adj2 absces*).ti,ab. or wound infection*.ti,ab.  AND  exp disinfectant agent/ or (solution or agent adj2 (antiseptic* or antimicrobial*)).ti,ab. or (skin adj2 (d?sinfectant or srub or preparation* or antisep* or detergent* or cleaning or cleansing)).ti,ab. or exp povidone iodine/ or exp iodophors/ or exp iodine/ or iodin*.ti,ab. or iodophor*.ti,ab. or Povidon-Iod*.ti,ab. or Betadona.ti,ab. or Brauno*.ti,ab. or Destrobac.ti,ab. or Jodoplex.ti,ab. or Pol?sept.ti,ab. or Sepso J.ti,ab. or Topionic.ti,ab. or Wundesin.ti,ab. or Betasept*.ti,ab. or 2 propanolol.ti,ab. or exp alcohol/ or ethanol*.ti,ab. or alcohol*.ti,ab. or IPA.ti,ab. or soluprep.ti,ab. or cutasept.ti,ab. or softasept.ti,ab. or exp chlorhexidine/ or Chlora?rep.ti,ab. or Chlorostat.ti,ab. or Hibi*.ti,ab.  AND  exp child/ or exp infant/ or exp adolescent/ or exp newborn/ or exp child health/ or exp pediatric hospital/ or exp intensive care units, neonatal/ or exp intensive care, neonatal/ or intensive care units, pediatric/ or (adolescen* or infan* or newborn* or (new ADJ born*) or baby or babies or neonat* or child* or boy* or girl* or minors or underag* or (under ADJ1 (age* or aging)) or juvenil* or pediatric* or paediatric* or suckling* OR PICU OR NICU OR PICUs OR NICUs).ti,ab.  AND  exp cohort analysis/ or exp longitudinal study/ or exp prospective study/ or exp follow up/ or cohort$.tw. or exp case control study/ or (case$ and control$).tw. or exp case study/ or (case$ and series).tw.  OR  exp crossover procedure/ or exp double-blind procedure/ or exp randomized controlled trial/ or exp single-blind procedure/ or (random* or factorial* or crossover* or cross adj1 over* or placebo* or doubl* adj1 blind* or singl* adj1 blind* or assign* or allocat* or volunteer*).ti, ab. |
| Cochrane CENTRAL | Child  AND  Surgical Wound Infection  AND  (Iodine Compounds or Chlorhexidine or Ethanol or Antiinfective agents, local) |
| CINAHL | ( surgical site infection or surgical wound infection or ssi )  AND  ( Antiinfective Agents, Local Administration and Dosage or Skin Preparation, Surgical or povidone iodine or povidone or betadine or iodine or povidone-iodine or chlorhexidine or chg or chlorhexidine gluconate or ethanol or alcohol )  AND  ( infant or child or pediatric or paediatric or children ) |
